# Paralysis Agitans

**Published:** 1909-08-14

**Authors:** Purves Stewart

**Affiliations:** Physician to Westminster Hospital and to the West End Hospital for Nervous Diseases


### PARALYSIS AGITANS.

By PURVES STEWART, M.D., F.R.C.P. Physician to Westminster Hospital and to the West End Hospital for Nervous Diseases.

(A Lecture delivered at the Medical Graduates' College and Polyclinic.)

Just as the ancient monuments of Egypt and Greece challenge comparison with any modern buildings, so also Parkinson's description of the disease called by his name—a description published nearly a century ago—remains a model of clinical accuracy and completeness. It is remarkable how little has been added to our knowledge of this malady since its original description in the year 1817. Paralysis agitans, or the "shaking palsy," as Parkinson termed it, is a disease of the central nervous system, whose anatomical substratum is as yet unknown, though the diagnosis can usually be made at a glance.

Look at this typical case in a man aged 51. Observe not only his tremors, but his gait as he walks in, and his posture when he stands still. Notice his bowed head, spiked on, as it were, to his stooping shoulders. See how cautiously he moves along, with short, shuffling steps, and notice particularly that his upper limbs, stiffly fixed at the shoulders, have lost the normal swinging movement of a healthy person, whilst the elbows are held semi-flexed, the wrists slightly dorsi-flexed, and the fingers and thumb in the "interosseal" posture, as in holding a pen, yet constantly trembling in a rhythmic fashion, as if rolling an invisible cigarette. We ask him to turn round, and he does this slowly and all in one piece, as if made of glass. We look at his face and see his staring eyes, their awestruck expression, with hardly a blink of the lids. His whole face looks stiff and frozen, and there is marked deficiency of facial expression when he speaks. We cover up his mouth while he talks, and see that the upper part of his face is practically immobile—the so-called "Parkinsonian mask." Now we offer him a chair, and notice how slowly and cautiously he sits down. Even when he sits, he seems as if he cannot let himself go, but appears always on the verge of getting up. Meanwhile, his toe rests on the ground, while his ankle trembles, synchronously with his shaking hand. We now ask him to get up again. He does so with great deliberation, and only succeeds after several preliminary failures.

Let us now consider the disease more in detail. As a matter of experience, more than four-fifths of the cases commence after the age of 40. But in exceptional cases the disease may come on in early adult life. I have seen it in a young man of 22, and Duchenne even described a case in a 17-year-old patient. Most cases come on gradually, but in a few the symptoms date from a fright or emotional disturbance. I remember the case of a man who was visiting a menagerie, when a lion broke loose from its cage and caused a panic in the audience: the first symptom of paralysis agitans was noticed next day in the form of stiffness of one leg. I have seen other cases attributed to such incidents as tooth-extraction, cab accidents, emotional shock, etc.; but dramatic incidents like these are quite exceptional, and I believe that in most cases sudden shock, if it has any influence, merely serves to exaggerate or render evident the disease which was previously latent.

The phenomena of the disease are mainly motor. The most obvious symptom is rhythmic tremor, of moderate speed (3 to 5 per second), more marked in the upper limbs than in the lower, and more marked at the distal than the proximal joints. The typical movement is sometimes limited to the thumb and index, which perform a pill-rolling or cigarette-rolling movement. Then some cases have flexion extension and abduction-adduction movements of all the fingers, and pronation-supination movements of the hand. The elbow and shoulder joints rarely show tremors, except for a communicated movement from the shaking hand. In the lower limbs the tremor is best marked at the ankle, and is synchronous with the digital tremor. The tremor of paralysis agitans is continuous, commencing a few moments after the patient wakes, and ceasing during sleep. Usually the patient can diminish or stop his tremor for a few seconds, in order to perform a voluntary movement—*i.e.* threading a needle. I remember a patient in a country village who had the most marked tremors, but if he was invited to console himself with a glass of whisky at the bar of the village hostelry his hand became as steady as a rock. But this rule is not invariable, for in a few cases the tremor is exaggerated by voluntary movement. Practically every case has a characteristic handwriting—slow, tremulous, and with an inclination to write smaller than usual. The lips, tongue, lower jaw, and vocal cords may be tremulous, but the respiratory and trunk muscles do not tremble. But although tremor is a very dramatic phenomenon in the disease, it is not essential. A certain proportion of cases have no tremors at all—so-called "paralysis agitans sine agitatione." In spite of the absence of tremors, we recognise these cases easily by their characteristic posture and gait, which are due to muscular rigidity.

Muscular rigidity indeed is the most constant of all the signs. We notice it in the limbs, which tend to be semi-flexed, also in the face, as already described, and in the muscles of the trunk and neck. Rigidity is also quite distinct when we move the patient's limbs passively. It causes a considerable degree of bodily discomfort, so that the patient frequently complains of difficulty in finding a comfortable position when lying in bed. Some patients have a peculiar difficulty in getting into bed. They may take several minutes to climb in, and when they have got into bed they feel themselves stiff and helpless. We have seen how slowly the patient turns round; we notice his gait, which tends to the "festinant" type—*i.e.* the steps become shorter and faster, and the patient may almost topple over, so-called propulsion. Similar appearances may be elicited if we ask the patient to walk backwards, when he tends to fall backwards—so-called retropulsion.

Some ten years ago I described a new symptom, previously unnoticed, which I called the "toe-curling phenomenon." This consists of a tonic spasm of the toe, usually flexor in type, less frequently extensor, which pulls the patient up when walking, so that he has to stand still until the toe relaxes. This symptom is often early in appearance, and occurs in about one-fourth of the cases.

However severe the case, there is never any true paralysis, although voluntary movement may be severely hampered by the muscular rigidity. Not only do the patients look downhearted, they also feel downhearted, and in a few cases melancholia may actually supervene.

The patient frequently complains of various subjective sensations, especially a feeling of continual restlessness, hot flushes, etc. Occasionally there is excessive sweating, and sometimes the patient complains of salivation. Salivation, however, may be explained in some cases as due to rigidity of the muscles of deglutition, whereby the saliva is retained a long time in the mouth.

Not infrequently the patient complains of frequency of micturition, and even of enuresis. In one of the patients whom I show you to-day enuresis was the earliest and most persistent symptom: Mr. Thomson Walker, who originally sent him to me, found no abnormality in his urinary tract to account for the symptom, which appears to be part of the nervous malady.

The course of the disease is steadily progressive. The malady does not shorten life, although it takes away much of the joy of living.

### Pathology Treatment.

Various theories have been propounded to account for the symptoms, but no constant anatomical changes have yet been demonstrated in the nervous system or elsewhere. And yet there must be a profound molecular disturbance somewhere, probably in the cerebrum. The hemiplegic onset of the disease points, of course, to some spot above the medulla, as the *fons et origo* of the malady. I cannot help feeling that we have to look to the mid-brain for the probable starting-point of the disease. Gross lesions in the neighbourhood of the red nucleus in the dorsal region of the crus cerebri produce rhythmic tremor of the contra-lateral arm and leg, and possibly paralysis agitans is the result of molecular change in that region.

Lastly, as to treatment. We must frankly confess that, although the patient's life is not shortened by the malady, cure is out of the question. The disease belongs to the degenerative group, and steadily becomes worse. But we can do something to alleviate the patient's discomfort. The chief means at our disposal consists in the administration of hyoscine in full doses, commencing with gr. 1/200 t.i.d., and cautiously increasing it. A few years ago formic acid was strongly recommended by some French observers, but I found that the remedy, even when greatly diluted, was so bitter that the patients said the cure was worse than the disease. Anyhow, I could not get them to persevere long enough with formic acid to produce any appreciable results. Warm baths and passive movements help to reduce the rigidity, so also does galvanism. Massage is of little use, whilst faradism makes the rigidity worse. Cheerful surroundings and encouraging suggestions also help to alleviate the patient's discomfort, and alleviation is all that we can attempt.

